# Preclinical assessment of the selective androgen receptor modulator RAD140 to increase muscle mass and bone mineral density

**DOI:** 10.14814/phy2.70463

**Published:** 2025-07-18

**Authors:** Jake Puskas, Teja Guda, Sarah Niccoli, Christopher R. Rathbone, Benjamin Tan‐Johnson, David Puskas, Ryan Middleton, Jeffrey S. Otis, Simon J. Lees

**Affiliations:** ^1^ Department of Biology Lakehead University Thunder Bay Ontario Canada; ^2^ Department of Biomedical Engineering and Chemical Engineering University of Texas at San Antonio San Antonio Texas USA; ^3^ Northern Ontario School of Medicine University Thunder Bay Ontario Canada; ^4^ Department of Kinesiology and Health Georgia State University Atlanta Georgia USA

**Keywords:** cross‐sectional area, functional overload, hypertrophy, RAD140, SARM, skeletal muscle

## Abstract

Selective androgen receptor modulators (SARMs) are important hypertrophic molecules that are potential treatments for many types of myopathy and osteopathy. This study aimed to determine if the SARM RAD140 had additive effects on muscle hypertrophy when combined with the functional overloading (FO) of the plantaris muscle. Male, Sprague–Dawley rats (*n* = 10 rats/group) were randomly selected into one of four treatment groups: (1) RAD140‐FO, (2) RAD140‐Control, (3) Vehicle‐Control, or (4) Vehicle‐FO. RAD140 groups received drugs through drinking water, and control groups received only Vehicle (methylcellulose). Standard rat chow and water were provided ad libitum. Muscle weights of the triceps‐surae group and muscle fiber cross‐sectional area (CSA) were measured. Muscle weight analysis showed a marked increase in RAD140‐FO groups but was not statistically different from the Vehicle‐FO group. CSA results indicated similar findings; however, RAD140‐Control showed significantly elevated CSA compared to Vehicle‐Control. The tibial microarchitecture was analyzed using micro computed tomography. RAD140 did not impact cortical or trabecular bone structural morphometric properties following 14 days of treatment. The data presented here show the potential of RAD140 to stimulate muscle hypertrophy in young healthy rats.

## Graphical abstract

Animals received a FO surgery and a contralateral sham surgery as a control. The effects of RAD 140 treatment were extensively evaluated for functional changes at the end of the 14‐day study.

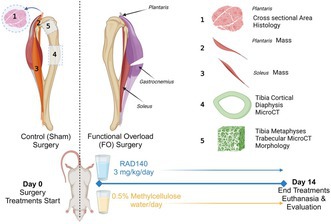

## 1 INTRODUCTION

Recently, researchers have been exploring using nonsteroidal selective androgen receptor modulators (SARMs) as a novel approach to androgen treatments. SARMs are novel pharmaceutical molecules developed to have a high affinity for the androgen receptors (AR) within muscle and bone tissue while displaying minimal downstream AR signaling effects within sex tissues such as the prostate or gonads (Narayanan et al., 2018). The AR is part of a large group of nuclear receptors in the body and includes receptors that bind estrogens, progesterone, glucocorticoids, mineralocorticoids, and androgens (Gao, Bohl, & Dalton, 2005). The AR is found in tissues such as skeletal muscle, cardiac and smooth muscle, bone, prostate, seminal vesicles, male/female genitalia, liver, skin, sebaceous glands, and the brain (Negro‐Vilar, 1999). The AR has a DNA and ligand‐binding domain activated via testosterone and dihydrotestosterone through the 5α‐reductase enzyme (Azhagiya Singam et al., 2019). However, other hormones, growth factors, and peptides can bind to the AR. A ligand must bind to an available receptor in the cytoplasm to activate the AR. The efficacy of a ligand (SARM or testosterone) in the activation process is based upon its ability to bind and replace the endogenous hormone typically activating the receptor (Azhagiya Singam et al., 2019). When the AR is inactive, its binding region is occupied by heat shock proteins (HSPs); however, in the presence of a ligand, these HSPs are unfolded and make room for the ligand to form the AR‐ligand complex. This binding allows the complex to translocate into the nucleus, where it binds to an androgen response element (ARE), which further regulates gene expression (Heemers & Tindall, 2007). Testosterone and SARMs are both ligands that produce anabolic responses on target tissues. Once bound, the ARE regulates the expression of mRNA related to protein synthesis. Tissue specificity of SARM‐induced signaling is based on the SARM‐AR complex formed within each tissue. These complexes are formed with various cofactors and regulatory proteins within the nucleus. While the exact mechanisms leading to tissue‐specific responses remain unclear, it is thought that the tissue‐specific regulatory milieu within different tissues leads to the tissue‐specific responses (Solomon et al., 2019). For example, the recruitment of cofactors and regulatory proteins to the SARM‐AR complex can be different in certain tissues, like the prostate, compared to the testosterone‐AR complex (Hikichi et al., 2015).

The development of these novel pharmaceuticals created an opportunity to combat conditions of skeletal muscle wasting while minimizing the adverse side effects commonly associated with androgen treatments or other anabolic androgenic steroids. While testosterone poses a promising solution to contest muscle‐wasting diseases such as seen in cancer cachexia (Srinath & Dobs, 2014), there are considerable downsides to the treatment as well. However, testosterone supplementation is associated with many adverse side effects (El Osta et al., 2016; Rolf & Nieschlag, 1998). Since testosterone is an endogenous hormone, it also affects sex tissues and can cause an enlargement in the prostate and seminal vesicles of men undergoing the treatment (El Osta et al., 2016). This may put individuals undergoing these treatments at an increased risk for the development of certain prostate‐related diseases. Several different SARMs are under investigation; some have shown promising results on muscle mass, bone mineral density, and functional tests (Fonseca et al., 2020). The common negative side effects from anabolic androgenic steroids have been proposed to be mitigated or reduced in SARM patients, which is where these novel pharmaceuticals make their case as the better alternative (Bhasin et al., 2006). SARM RAD140 is highly tissue selective with a high affinity for the anabolic AR on skeletal muscle (Miller et al., 2011). These factors make RAD140 an excellent candidate for possible future clinical use. Due to the need for more understanding surrounding these novel drugs, extensive research must be completed to determine the efficacy and safety of the SARM RAD140 before any human applications are possible.

SARMs have been previously investigated for their ability to offset loss of bone and muscle mass in orchiectomized (ORX) rats (Gao, Reiser, et al., 2005; Jones et al., 2009). In addition, clinical trials have been conducted to explore the potential benefit of SARM therapy in cancer cachexia (Crawford et al., 2016; Dobs et al., 2013). In addition, there would be a potential benefit of SARM therapy within the context of age‐related skeletal muscle loss (sarcopenia) and bone loss (osteopenia and osteoporosis), as well as skeletal muscle loss as a result of prolonged bed rest or space flight. One area of interest for the clinical use of SARMs involves the combination of their pharmaceutical use with resistance exercise aimed at increasing skeletal muscle size and strength. The current research sought to determine if RAD140 improves muscle growth and bone mineral density/microarchitecture in combination with a resistance exercise model. These results may serve as a basis for this approach to be applied in a clinical setting to combine a training program with RAD140 supplementation treatments (e.g., prehabilitation and rehabilitation). In preclinical models, FO is a resistance training model that induces animal muscular hypertrophy. This model works to achieve mechanical overload by chronically increasing the load on an animal's muscle group(s) by surgical intervention through removing critical synergist muscle(s) (or distal portions of muscle(s)), leaving the remaining muscle(s) to maintain the animal's posture and mobility (Terada et al., 2012; Terena et al., 2017). The FO model, combined with testosterone treatments, has been proven to significantly increase muscle mass and the cross‐sectional area of myofibers. Previous research has reported that the SARMs MK‐0773, GTx‐024, and LGD‐4033 can increase lean body mass and bone mineral density (BMD). There is also a link between resistance training and the FO model to bone remodeling. Resistance training in humans and FO in rodents are known to cause increases in various circulating factors, including IGF‐I, growth hormone, and testosterone (Zouhal et al., 2022). Some of these skeletal muscle secretome elements have been linked to increasing bone mineralization (Leuchtmann et al., 2021). For example, IGF‐1 has been demonstrated to increase bone mineralization (Breen et al., 2011) and bone growth (Banu et al., 2003). In addition, the FO model could influence other less well‐described elements of the secretome that could impact bone mineral density (Leuchtmann et al., 2021). To date, no investigation has been conducted into using the SARM RAD140 in combination with this resistance training model. In the current study, we tested whether RAD140 works in combination with FO to significantly increase muscle hypertrophy while maintaining/improving bone mineral density and microarchitecture in the functional overload model in rats.

## 2 MATERIALS AND METHODS

### 2.1 Functional overload model in vivo

All procedures were approved by the Lakehead University Animal Care Committee and the Georgia State University Institutional Animal Care and Use Committee. Male Sprague–Dawley rats (ordered at 226–250 g, *n* = 10 rats/group) were purchased from Charles River Laboratories and housed under a 12:12 light–dark cycle at room temperature in the Georgia State University animal facility, which maintains a strict temperature and humidity range ideal for rodent housing. Animals were housed in the Georgia State University Animal Resources and fed a standard pelleted diet from Harlan Teklad (Indianapolis, IN, USA) ad libitum. They were housed in polycarbonate shoebox cages with a ventilated lid and sterile watering system. Animals are housed 2 per cage and provided enrichment (e.g, tunnels). Animals were given 1 week to equilibrate to the new environment before starting the experiment. Rats were provided free access to food and water during this time. They were then randomly assigned to one of four groups (*n* = 10/group): Group 1: functional overload (FO) surgery + Vehicle (0.5% methylcellulose) (Vehicle‐FO), Group 2: FO surgery + RAD140 in 0.5% methylcellulose (RAD140‐FO), Group 3: Control surgery + Vehicle (0.5% methylcellulose) (Vehicle‐Control), and Group 4: Control surgery + RAD140 in 0.5% methylcellulose (RAD140‐Control). RAD140 was obtained from SelleckChem (Houston, TX, USA, product number S5275.) and methylcellulose was obtained from Sigma Aldrich (St. Louis, MO, USA, product number M0512). Once rats were assigned to individual treatment groups, functional overload (FO) surgery was performed on one limb, preserving the non‐operated leg to serve as an internal control for hypertrophy, which helps to minimize variability and also allows us to reduce the number of animals required. For the FO, the distal tendon of the gastrocnemius muscle was isolated from the tendons of its major synergists (soleus and plantaris) and transected along with the distal portion of the muscle. Only the very distal portion of the muscle was removed with the tendon, which would minimize the impact of surgery and account for a small portion of total body weight.

Following surgical intervention, rats received their first dose of RAD140 or methylcellulose. The RAD140 groups received RAD140 in their drinking water for two consecutive weeks. The target dose of 3 mg/kg/day was chosen because of the physiological relevance of the human equivalent dose (Gao, Reiser, et al., 2005; Miller et al., 2011). The water bottles were weighed at each bottle change to determine the estimated water consumption for each cage. The average daily water consumption for the entire 2 weeks was then determined for the estimated daily RAD140 doses by cage (mg/kg/day) (Table 1). The measurement of water consumption and the human equivalent dose (HED) estimation, which utilizes a correction factor (k_m_) that is estimated by dividing the average body weight (kg) of species by its body surface area (m^2^), was used to enable the conversion of doses from mg/kg to mg/m^2^. The conversion of doses normalized to estimates of surface area helps to account for differences in metabolic rates. By utilizing the K_m_ ratio of humans (K_m(human)_ = 37) to rats (K_m(rat)_ = 8.5) calculation, the dose attained in the rats can be converted to a HED by dividing by 4.35, which is the K_m_ ratio of humans to rats (adjusted for the rat size in this study) (Nair & Jacob, 2016). Using this estimation, an HED dose of 0.49 mg/kg/day was attained. In parallel subgroups of vehicle‐treated, rats received 0.5% methylcellulose in their drinking water. Water consumption stayed within expected ranges for all rats throughout the course of the study.

**TABLE 1:** Estimated daily RAD140 doses by cage (mg/kg/day) and the corresponding human equivalent dose (HED).

Cage	Average water consumption (g)	Average pre/post (kg)	Estimated dose (mg/kg/day)	HED dose (mg/kg)
I	33.77	0.46	1.84	0.42
II	42.16	0.42	2.48	0.57
III	34.82	0.42	2.08	0.48
IV	38.60	0.43	2.24	0.51
V	34.82	0.45	1.94	0.45

Unilateral, FO surgery was performed as previously described (Otis et al., 2007). Briefly, rats were deeply anesthetized (isoflurane: 5% induction at 1000 mL/min of oxygen followed by a maintenance dose of 1%–3% at 500 mL of oxygen), and a midline incision was made through the skin on the posterior aspect of the animal's lower leg. The distal tendon of the gastrocnemius muscle was isolated from the tendons of its major synergists (soleus and plantaris) and transected along with the distal portion of the muscle. Removal was done with particular care not to interrupt vasculature or innervation leading to the soleus or plantaris muscles. 3–0 Ethilon sutures were used to close the wound and were removed 10–14 days post‐surgery if not already removed by the animal. The incision was treated with a topical antibiotic (Bacitracin). The animals were then closely monitored for sudden changes in health following the surgical procedure. Surgery occurred on one hind limb, preserving the non‐operated leg as an internal control for plantaris hypertrophy.

All study animals were euthanized using CO_2_ inhalation followed by removal of the diaphragm 14 days after surgery. Skeletal muscle and bone samples were obtained after the animals were euthanized. The three hindlimb muscles of the posterior compartment (soleus, plantaris, and gastrocnemius) were removed, trimmed free of connective tissues and fat, blotted dry and weighed, and flash frozen in isopentane cooled in liquid nitrogen until future analyses. Tibias were removed, length measurements were taken, and they were fixed in 10% neutral buffered formalin before being shipped to the Central Animal Core Imaging and Transgenic Facilities at the University of Manitoba.

### 2.2 Muscle weights and plantaris cross‐sectional area

Plantaris muscles were removed, embedded in optimal cutting temperature compound (OCT), and immediately frozen in isopentane cooled in liquid nitrogen. The plantaris muscles were cut in 10 μm serial sections maintained at −20°C using a cryostat (CM1850, Leica Biosystems, Deer Park, IL). Sections were then processed for hematoxylin and eosin staining (H&E), dehydrated, mounted, and visualized at 20X with a Leica microscope. Cross‐sectional areas of approximately 50 fibers per muscle were calculated using ImageJ software (NIH, Bethesda, MD). One member of the investigation team was blinded to the groups and analyzed the 50 fibers per sample, across 2–3 fields selected at random. Unpublished observations from our group have found little change to the overall fiber area average with more fibers analyzed, and our group has previously published with fewer than 50 analyzed fibers (Otis et al., 2004).

### 2.3 Micro computed tomography

The tibia collected from the animals was all scanned using a SkyScan 1176 (Bruker Biospin, Billerica, MA) at a source voltage of 50 kV and tube current of 500 μA, to acquire images at a resolution of 8.89 μm, which were then reconstructed using NRecon (Bruker Belgium, Kontich, Belgium). The image stack of reconstructed images was then realigned such that the principal vertical axis was aligned with the longitudinal axis of the tibia. Two volumes of interest (VOI) were selected for analysis, one in the metaphysis and the other in the midshaft regions of the femur. In the tibial metaphysis, the trabecular bone VOI was extracted beginning at the growth plate and extended 300 slices (2.67 mm thick). Binary bitwise operations were used to identify the perimeter of the cortical bone at the endosteal boundary and automated contours created to separate the trabecular volume in this VOI. A grayscale value threshold of 84 was determined using the Otsu algorithm to threshold across the entire study to identify mineralized tissues. The analysis of cortical bone structure was performed over 600 slices (5.33 mm thick) centered at the 50% position (from proximal to distal) in the tibial diaphysis. The cortex was selected using automated, density‐driven contouring with the threshold maintained at 84. Bone microarchitecture was evaluated by determining various structural parameters from the Micro Computed Tomography (microCT), such as cortical and trabecular bone volume fractions, cortical bone mineral density, cortical mean polar moment of inertia, trabecular bone surface to volume ratio, trabecular connectivity density, trabecular spacing, trabecular number, and trabecular thickness. The structural morphometric properties of the cortical and trabecular regions were analyzed using CT Analyser software (CTAn 1.18.8.0, Bruker Skyscan).

### 2.4 Statistical analysis

Comparisons between treatment groups were made using Student's *t*‐test (for water consumption) or two‐way ANOVA (for body weight, plantaris muscle fiber cross sectional area and muscle weights, using a generalized linear model to identify differences across the treatment type and surgery type). All datasets met the Shapiro–Wilk test for normality and the Brown‐Forsythe test for equal variance and thus the parametric ANOVA models were evaluated. Pearson's correlation coefficients (*r*) and corresponding coefficients of determination were calculated for estimated RAD140 consumption and the following variables: plantaris CSA, plantaris muscle mass, and soleus muscle mass. Significant differences were determined at the *p* < 0.05 level, and post hoc identification of groups with significant differences was performed using either Tukey's or Holm tests (for repeated measures ANOVA involving pre‐ vs. post‐intervention body weight). Statistical analysis was conducted using SigmaPlot (Grafiti LLC, Palo Alto, CA) and visualized using Prism (v9.3.1, GraphPad Software, Boston, MA).

## 3 RESULTS

### 3.1 Functional overload model and water consumption dosing

**FIGURE 1:**
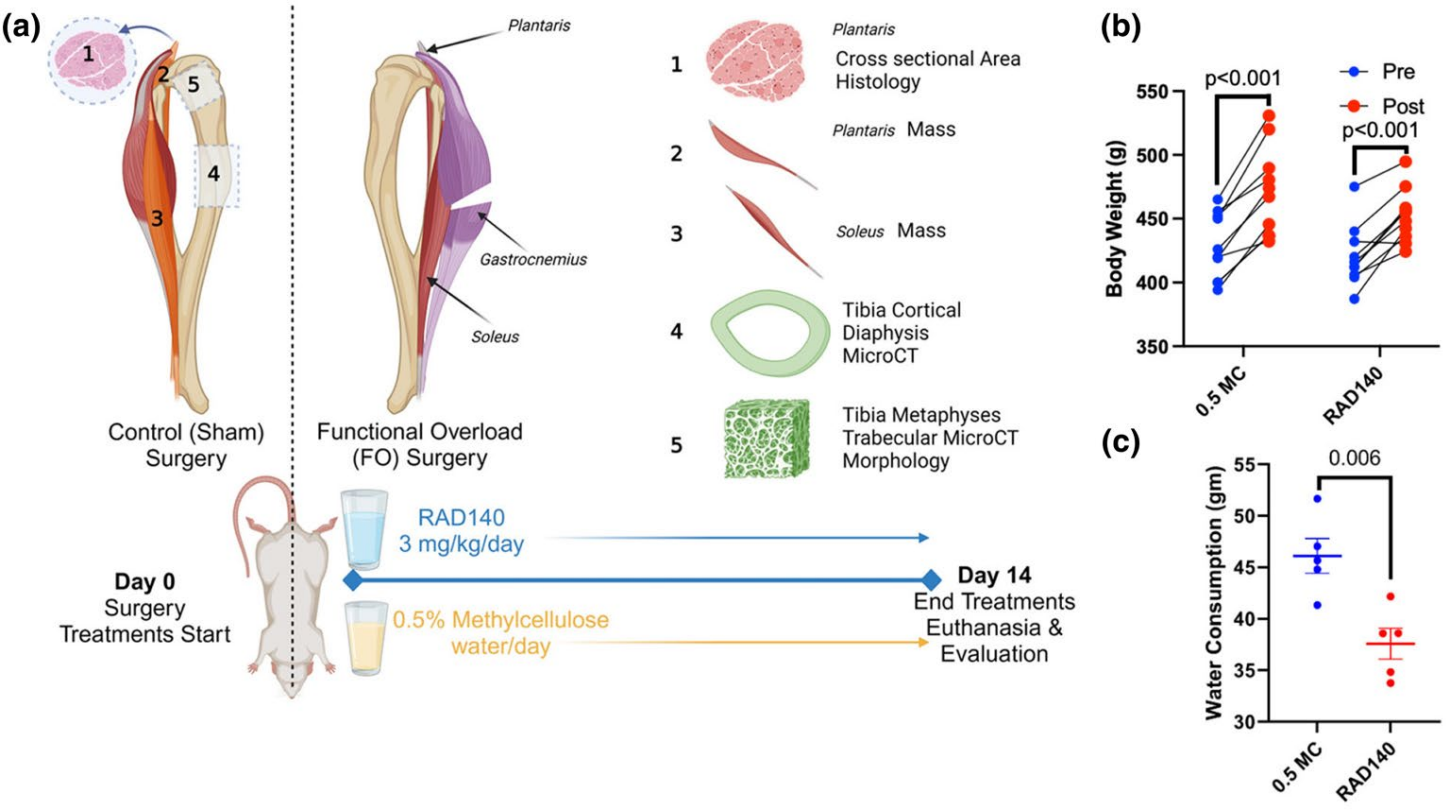
(a) Schematic of the overall study design—each animal received a functional overload surgery by gastrocnemius bisection and a contralateral sham surgery as control. Half the animals were treated with RAD140 + 0.5% methylcellulose (RAD140) and half with control 0.5% methylcellulose (0.5 MC) as control in their daily water intake. All groups were extensively evaluated for functional changes at the end of the 14‐day study (b) Relative change in body weight plot highlighting the comparison of post‐intervention body weights of 0.5% methylcellulose and RAD140 treatment groups ± SD. The groups were not statistically different (ns). (c) Average daily water consumption (gm) per cage by treatment group ± SD. Statistical significance is indicated by the *p* value for water consumption.

Body weights were taken as soon as the rats arrived at the animal facility, and they were given ample time to acclimate. At the time of euthanasia, body weights were retaken. At the time of tissue collection, the skeletal muscle (plantaris and soleus), and bone (tibia) were collected from the sham limb and contralateral FO limb for cross‐sectional histology and microCT (Figure 1a). Body weights from the rats in the methylcellulose as well as the RAD140 treatment groups were significantly higher than their weights measured at the beginning of the study. Body weights were not statistically different between Vehicle and RAD140 either pre‐ or post‐intervention (*p* = 0.496 and *p* = 0.305, respectively). Both the Vehicle (*p* < 0.001) and RAD140 (*p* < 0.001) groups demonstrated a significant increase in body weight pre‐ versus post‐intervention (Figure 1b).

Animals were pair‐housed for health purposes, which should be considered when viewing these data. Water weight measurements were taken each time the treatment water needed to be filled. This occurred roughly five times per cage ± one refill throughout the 14‐day study. A standard animal cage water bottle was weighed empty, and then 500 g of treatment solution was added. Measurements taken on a fill day were then used with measurements taken on a refill day to determine the quantity of treatment water consumed. This was then averaged across the period from the first measurement to give a daily average per cage, which was used to estimate consumption for each animal. The methylcellulose group drank significantly more water than the RAD140 group (*p* = 0.006) (Figure 1c).

Using the average water consumption by cage and the average weight per cage, an estimated daily dose of RAD140 was calculated (mg/kg/day). Our original target dose was 3 mg/kg/day; however, the estimated amount of RAD140 consumed was between 1.8 and 2.5 mg/kg/day per animal (Table 1).

### 3.2 FO and RAD140 effects on muscle mass and cross‐sectional area

**FIGURE 2:**
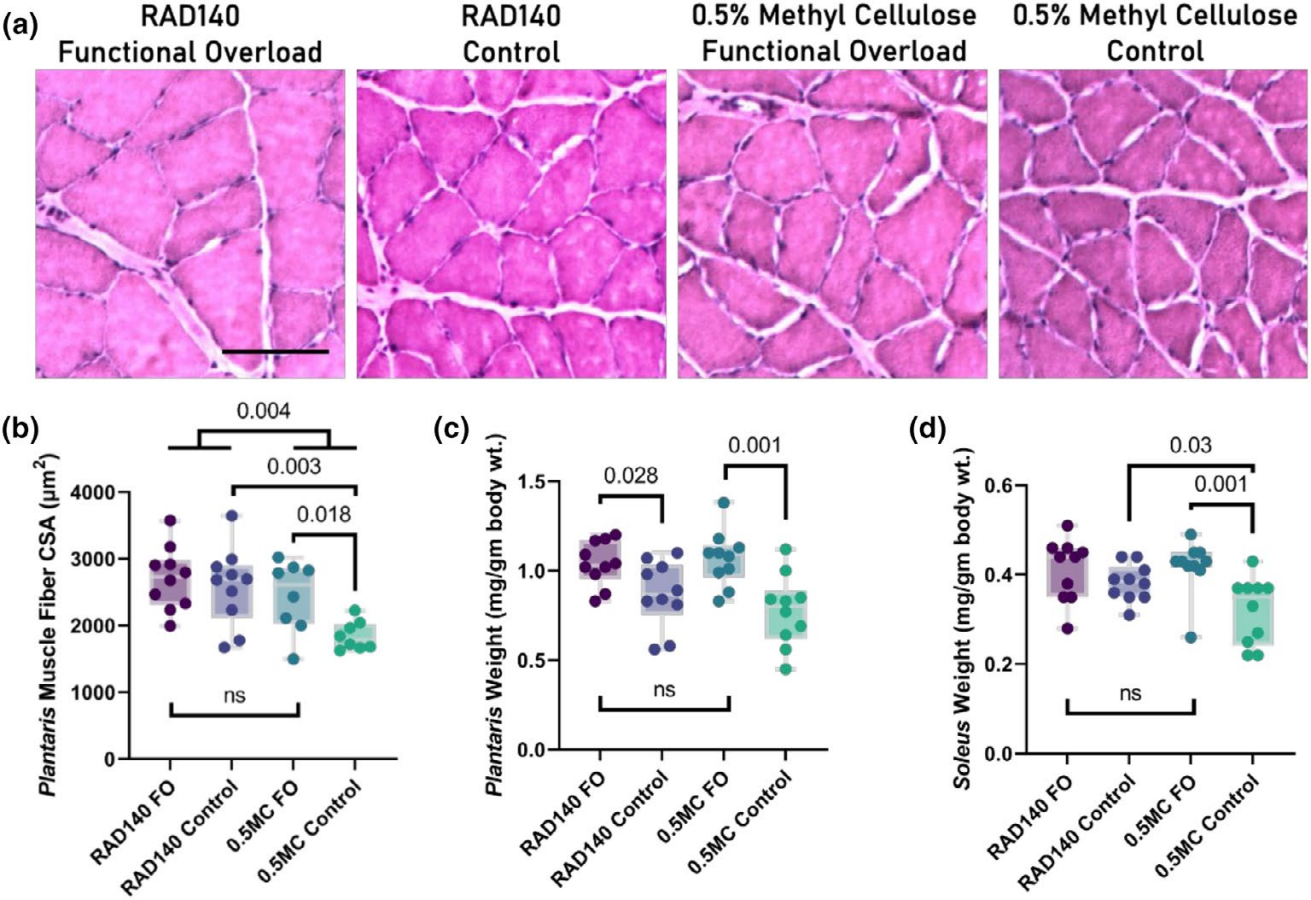
(a) Representative histology of rat plantaris muscles: RAD140 + 0.5% methylcellulose (RAD140) and vehicle 0.5% methylcellulose (0.5 MC) from the functional overloaded (FO) limb or the contralateral control (Control). Magnification bar indicates 100 μm. Box and whisker plots illustrating the (b) plantaris cross‐sectional area (μm^2^), normalized weights of the (c) plantaris and (d) soleus muscles (mg/gm of total body weight) at study termination, where the limits of the box are the upper and lower quartiles, the whiskers are the upper and lower extremes, and the line within the box is at the median. Statistical significance is indicated by the *p* values for the comparisons; ns indicates a nonsignificant difference.

The FO model causes plantaris hypertrophy by forcing the muscle to support body weight due to the absence of the gastrocnemius muscle. Plantaris muscles were harvested at euthanasia, blotted dry, weighed, and then flash frozen. Muscle hypertrophy can be gauged by increases in muscle mass and cross‐sectional area. Tissue sections of the plantaris muscle were evaluated for cross‐sectional analyses of fiber size. Representative images for tissue cross sections for each treatment group are presented in Figure 2a. There was a main effect of CSA with both administration of RAD140 (*p* = 0.004) as well as FO surgery with an increase compared to control surgery (*p* = 0.032) (Figure 2b). The RAD140‐FO group was not statistically significant compared to the RAD140‐Control group (*p* = 0.571). The Vehicle‐FO group demonstrated a statistically significant increase in plantaris CSA compared to the Vehicle‐Control group (*p* = 0.018).

The plantaris muscle masses were significantly increased (*p* < 0.001) with FO surgery compared to the contralateral control limbs, but there was no significant difference between RAD140 administration compared to vehicle within FO (*p* = 0.562) (Figure 2c). The plantaris muscle mass was statistically greater in the RAD140‐FO group, compared to the RAD140‐Control (*p* = 0.028) (Figure 2c). The FO model was designed to maximize hypertrophy in the plantaris muscle; however, this model also creates additional load for the surrounding intact muscles of the triceps surae group. This includes the soleus muscle, which was left intact during our surgical intervention. Soleus muscle mass, normalized to body mass is presented as a box plot (Figure 2d). Soleus muscle weights were significantly different (*p* = 0.002) between the FO and contralateral control groups, with no significant impact of RAD140 administration (*p* = 0.736) in the FO surgery, but a significant increase in soleus weight with RAD140 administration in the contralateral control groups (*p* = 0.03). There were no significant differences between any of the treatments (surgery or drug administration) on the weight of the gastrocnemius (*p* > 0.244, data not shown). Correlational analysis was performed to determine if any relationships existed between the estimated RAD140 dose and plantaris muscle fiber CSA, plantaris weight normalized to body weight, and soleus weight normalized to body weight (Table 2). Only the soleus weight normalized to body weight demonstrated a significant relationship to the estimated RAD140 dose (*p* = 0.010).

**TABLE 2:** Correlational analysis of the estimated RAD140 dose by cage and the measurements of skeletal muscle hypertrophy.

Variable correlated against estimated RAD140 dose	Pearson's correlation coefficient (*r*)	Coefficient of determination (*r* ^2^)	*p* Value
RAD140‐control			
Plantaris CSA	−0.172	0.030	0.634
Plantaris muscle mass (mg/gm body wt.)	−0.483	0.233	0.158
Soleus muscle mass (mg/gm body wt. g/kg BW)	−0.344	0.112	0.346
RAD140‐FO			
Plantaris CSA	−0.397	0.158	0.257
Plantaris muscle mass (mg/gm body wt.)	−0.072	0.005	0.842
Soleus muscle mass (mg/gm body wt.)	−0.766	0.587	0.010

### 3.3 FO and RAD140 effects on mid‐diphyseal cortical and metaphyseal trabecular bone

**FIGURE 3:**
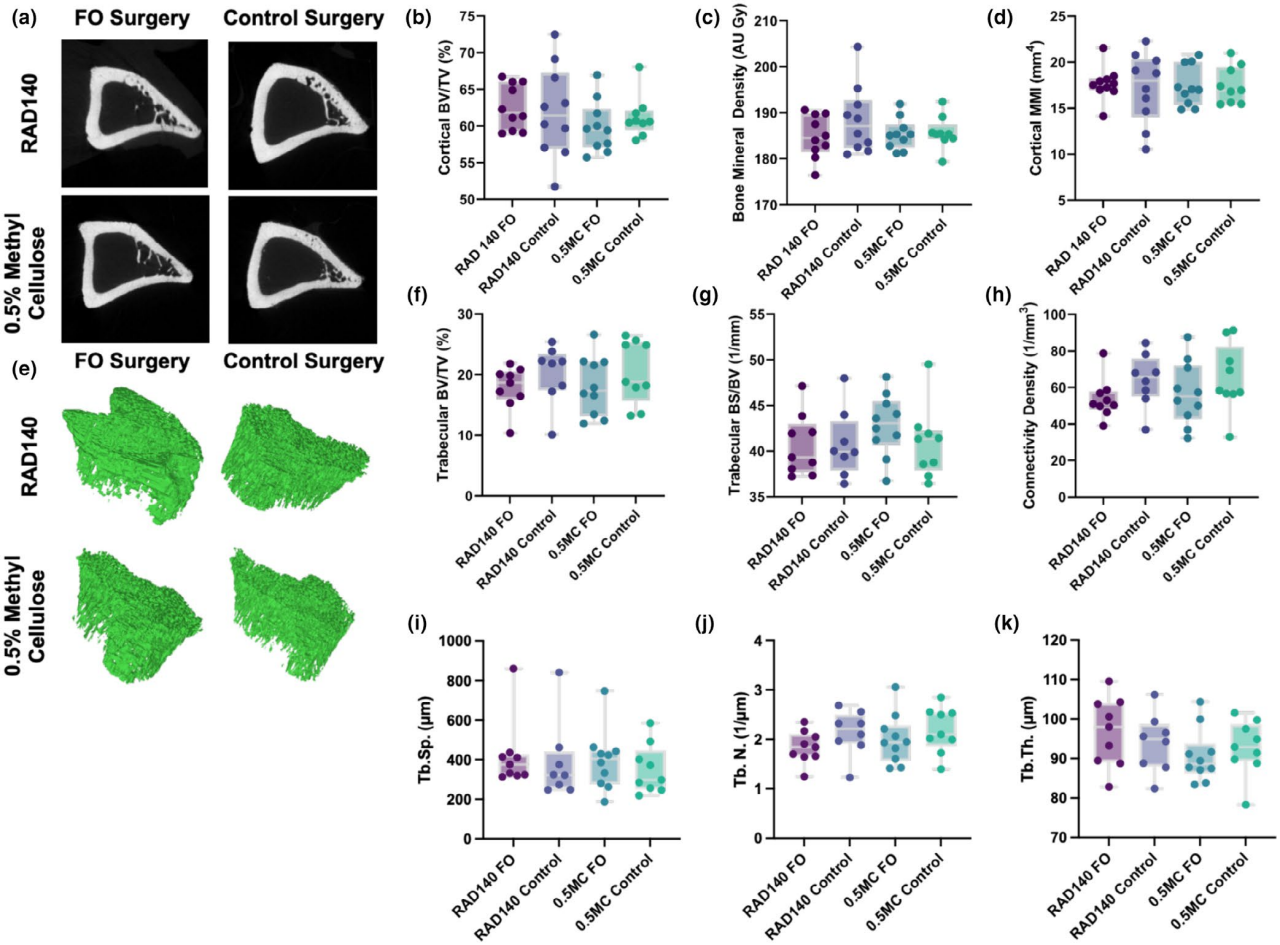
Representative microCT reconstructions of the (a) diaphyseal cortical bone and (e) epiphyseal trabecular bone from the tibia for each treatment group. The cortical (b) bone volume fraction (BV/TV) (%), (c) bone mineral density (grayscale AU), and (d) polar mass moment of inertia (MMI) (mm^4^) were compared between groups. Trabecular morphometry was conducted by computing the trabecular (f) bone volume fraction (BV/TV) (%), (g) bone surface to volume ratio (BS/BV) (mm^−1^), (h) connectivity density (mm^−3^), (i) trabecular spacing (Tb.Sp.) (μm), (j) trabecular number (Tb.N.) (1/μm), and (k) trabecular thickness (Tb.Th.) (μm). For the box and whisker plots, the limits of the box represent the upper and lower quartiles, the whiskers are the upper and lower extremes, and the line within the box is at the median. No significant volumetric, density, or morphometric differences were observed between treatment groups.

The FO model was designed to maximize hypertrophy in the triceps‐surae muscle group; however, this model also provided the opportunity to examine the effects of RAD140 on the underlying bone tissue. The reconstructed representative cortical and trabecular VOIs are shown for each of the four treatment groups in Figure 3a,e, respectively. No significant changes across treatment groups in cortical bone cross‐sectional thickness (*p* = 0.425), cross‐sectional area (*p* = 0.584), or cortical bone volume fraction (*p* = 0.5289, Figure 3b) were observed, indicating that there was limited impact on cortical bone quantity (Minonzio et al., 2018). Cortical bone mineral density (BMD) may indicate loss of calcium in bone tissue, typically endosteally when there is unloading (Minonzio et al., 2018). It was measured using microCT imaging with no statistically significant differences observed (*p* = 0.403, Figure 3c), indicating lack of change in bone quality. The cortical polar moment of inertia (MMI), an excellent measure for the structural integrity and ability to carry axial load or torsion of bone (Nair & Jacob, 2016), was measured using microCT imaging on the diaphysis of the tibia. No statistically significant trends were found in the data (*p* = 0.9796), indicating no changes in mechanical integrity (Figure 3d). Representative microCT images of each treatment group's distal metaphysis of the tibia were taken and visually analyzed for differences. Images were captured using a microCT imager, and no noticeable visual differences were evident between treatment groups.

The trabecular architectural data represents a region of bone extracted from the trabecular regions below the subchondral growth plate in the epiphysis of the tibia. The trabecular number is an essential physiological measure as it represents the number of trabeculae present and gives insight into the amount of remodeling inside the trabecular bone tissue (Minonzio et al., 2018). There were no statistically significant findings between treatment groups (*p* = 0.376, data not shown). Trabecular bone volume (BV%) is an important indicator as it represents the overall volume and connectivity of the trabecular bone (Hahn et al., 1992; Hipsley et al., 2020). There were no statistically significant findings (*p* = 0.547, Figure 3f).

Trabecular bone surface density measures the segmented bone surface ratio to the region's total volume of interest (Hahn et al., 1992). There were no significant differences in trabecular bone surface density measured in the tibial epiphyses (*p* = 0.534, Figure 3g). Trabecular pattern factor (TBPf) is the ratio of concave to convex surfaces of the bone pattern observed in two‐dimensional sections representing a three‐dimensional space (Hahn et al., 1992; Hipsley et al., 2020). Changes in osteoclast activity usually result in a noticeable change in TbPf, but no significant differences were found (*p* = 0.303). The trabecular structural model index (SMI) quantifies the number of rods and plates inside trabecular bone (Salmon et al., 2015), and the connectivity density is the number of connections between trabeculae per unit volume; no significant differences in trabecular connectivity density were observed, indicating that there was no significant resorption or accretion of trabecular bone mass or morphological changes (*p* = 0.110, Figure 3h). Regarding the trabecular architectural indices (Guda et al., 2014), trabecular spacing, which indicates the relative length of trabeculae and is equivalent to the size of the trabecular pores, showed negative correlation trends with trabecular thickness, which is the diameter of the individual trabecular struts, as is anticipated in bone physiology (Chen et al., 2017). However, there were no significant changes observed in trabecular spacing (*p* = 0.831, Figure 3i), trabecular number (*p* = 0.376, Figure 3k), trabecular thickness (*p* = 0.369, Figure 3k) or between any of the treatments, indicating that there was no impact on the trabecular architecture: trabecular number (Figure 3j) and trabecular thickness.

## 4 DISCUSSION

This study examined the effects of the SARM RAD140 combined with an FO model for hypertrophy. The hypothesis was that RAD140 and FO would independently result in hypertrophy but, in combination, would result in a more significant hypertrophic effect than any one treatment alone. To our knowledge, this is the first study to investigate the potential additive hypertrophic effects of both RAD140 and FO. In addition, this is the first study to investigate the potential bone remodeling effects of RAD140 using microCT. The outcomes from this study will help inform potential clinical applications for RAD140 in humans. Based on the muscle mass comparisons, RAD140‐FO caused an increase in plantaris muscular mass, compared to the contralateral control (RAD140 alone). However, plantaris mass for RAD140‐FO was not different from the vehicle‐FO group. RAD140 alone increased plantaris muscle fiber cross‐sectional area; however, there was no difference between RAD140‐FO and the vehicle‐FO group. Similar results were found in the soleus muscle. Soleus mass was greater in the RAD140 alone, compared to the Vehicle‐Control; however, there was no difference between the Vehicle‐FO and either the RAD140‐Control or the RAD140‐FO groups. Taken together, our data indicate that there is not a combination effect of RAD140 and FO; however, RAD140 alone has a similar hypertrophic effect as the FO model.

While it was expected that the rats would not consume identical amounts of water, it was assumed that they would consume an average daily quantity based on their body weights (Sharp & Villano, 2013). The concentration of RAD140 in 0.5% methylcellulose prepared for the treatment group was calculated to achieve a daily dose of ~3 mg/kg. The actual water consumption compared to predicted volumes resulted in the estimated daily doses consumed being lower than intended (Table 1). The estimated daily doses of RAD140 were 1.8–2.5 mg/kg/day. The estimated RAD140 dose should be considered in the interpretation of our findings. The use of paired housing of the rats was based on their well‐being, as rats are social animals. A correlational analysis was performed using the estimated RAD140 consumption for each cage against the average muscle mass of the plantaris and soleus, as well as the plantaris CSA for the animals housed in each cage. There was a significant correlation between estimated RAD140 consumption and soleus muscle mass normalized to body weight in the RAD140‐FO group (Table 2). We acknowledge there are problems with this analysis. First, we are unable to provide a correlative analysis of the dosage of RAD140 for each animal with the measurements of hypertrophy due to the paired housing of the animals. Second, due to the group housing, there were only 5 cages, which is a very small sample size for a correlation analysis (Aggarwal & Ranganathan, 2016). Finally, there was limited variability in the data with coefficients of variation of 11.9% in estimated RAD140 consumption and 17.4% in plantaris CSA in the RAD140‐FO group, which impacts the correlational analysis. However, it is possible that a higher a dose of RAD140 may result in an additive effect in combination with FO and could potentially cause measurable changes to bone remodeling.

Water consumption was measured for both treatment groups, and it was determined that the rats preferred to drink the 0.5% methylcellulose solution over the RAD140 + 0.5% methylcellulose (Figure 1c). Methylcellulose is one of the most commonly used vehicles in preclinical studies to aid in the suspension of insoluble treatments. The doses used have been previously investigated for nonclinical use as a vehicle (Gad et al., 2016). At 0.5%, methylcellulose is well tolerated with no adverse findings reported. However, higher concentrations of methylcellulose have been shown to increase water consumption (increased at 1% day in cats (Hall et al., 2021)). Since the rats drank significantly more of the Vehicle compared to the RAD140 (*p* = 0.0054), we can assume that there may be an attraction to the taste of the 0.5% methylcellulose alone or that there may be a deterrent associated with the smell or taste of the RAD140. Although the vehicle group consumed more water on average daily, the range at which they consumed it was still within normal levels (Sharp & Villano, 2013).

Functional overload is a surgical model designed to chronically load a muscle, resulting in hypertrophy for the remaining intact muscle or muscle group (Terada et al., 2012; Terena et al., 2017). This surgical procedure forces the animal to carry the same load with fewer muscle groups, thus increasing the load on the individual muscle(s). While the FO model was expected to induce sufficient muscle hypertrophy, the effects of a combined treatment between a muscle hypertrophy‐inducing model and a SARM, as well as, the effect of RAD140 on the contralateral/unloaded control were unknown. While it was hypothesized to have an additive effect, this was not the result in this study. Through statistical investigation of pre‐ and post‐body weights, it was evident that each group had significantly increased their pre‐intervention body mass (*p* ≤ 0.001—methyl, *p* ≤ 0.001—RAD140). However, the post‐intervention body masses for each treatment group were not statistically different from one another (*p* = 0.305). Therefore, the combination of RAD140 and FO did not have an additive effect more remarkable than that of RAD140 or FO alone on the overall body weights of the animals following the intervention. Relative to pre‐intervention, the observed change in each group was not statistically different between groups. While we have no evidence of an additive effect between RAD140 and FO, it is possible that prolonging the study from 14 days to 21 days may have given the RAD140 enough time to build up a more significant effect on muscle mass.

Further investigation into the individual muscles affected by the FO model revealed that FO and RAD140 caused an increase in overall muscle mass of the plantaris muscle (Figure 2) compared to the Vehicle‐Control; however, these groups were not statistically significantly different. The RAD140‐Control group was not statistically different from any other group. Due to the robust nature of the FO model, the effect of the FO model may have masked the effect of the RAD140 in such a short time frame.

The plantaris muscle is not the only muscle group responsible for carrying the increased load in our FO model. Although it is the primary load‐bearing muscle of the group, the soleus muscle also bears a certain percentage of the body weight. It is also affected by the FO model. Investigation into the soleus muscle revealed that the RAD140‐FO and vehicle‐FO groups were statistically significant compared to the vehicle control group (*p* = 0.00376) but were not statistically different from the RAD140‐control group (Figure 2). Again, the same pattern arises as seen in the plantaris muscle. Due to the timeline of the intervention, the RAD140 groups may have needed more time beyond the 14 days to overcome the effects of the FO treatment, thus masking the actual effectiveness of the SARM itself. As both processes occur, it is possible that with such a robust intervention like FO, which has been proven to cause hypertrophy within a week of intervention (Bhasin et al., 2006; Lee et al., 2003; Rolf & Nieschlag, 1998; Washington et al., 2014), it may be difficult to reveal a potential combined effect of RAD140.

Previous results have demonstrated an increase in plantaris muscle mass and CSA with FO (Bigard et al., 2001; Thomson & Gordon, 2005). RAD140‐induced hypertrophy has been reported in the levator ani muscle in ORX rats in a dose‐dependent manner (Miller et al., 2011). However, the effects of RAD140 in both the intact/non‐castrated rat model, as well as, the potential additive effect of FO and RAD140 on plantaris and soleus muscle have not been investigated. The RAD140‐FO group and RAD140‐Control group were significantly different compared to the Vehicle‐Control group (Figure 2). However, the two RAD140 groups were not statistically different from the Vehicle‐FO treatment group. Additionally, the Vehicle‐control group was not statistically different from the Vehicle‐FO group (Figure 2). This indicates that RAD140, in combination with FO treatment, does not elicit a greater effect on hypertrophy compared to RAD140 alone.

SARMs have been found to act on the ARs located primarily within muscle and bone (Christiansen et al., 2020; Fonseca et al., 2020; Hoffmann et al., 2023; Liu et al., 2024; MacKrell et al., 2015; Miller et al., 2011; Zhang & Sui, 2013). Understanding the full range of effects these novel pharmaceuticals may possess is vital in creating positive change for osteopathy. The SARM RAD140 has previously been highly active in the AR of muscle tissue (Fonseca et al., 2020; Liu et al., 2024; Miller et al., 2011; Zhang & Sui, 2013). However, little information exists on its potential effects on the ARs inside the cortical and trabecular bone tissues. The bone formation rate and bone turnover (bone remodeling periods) are quite different between rats and humans. These rates are also affected by age and growth stage. The rats used in the present study were ~400 g at the beginning of the study, which corresponds to ~9–10 weeks of age, which represents an age of continued growth. Bone formation rates normalized to bone volume (BFR/BV) have been reported to be several times higher in rats compared to humans (Rubin et al., 2001; Taguchi & Lopez, 2021; Tanizawa et al., 1999). Bone remodeling periods, which is the average total duration of a single cycle of bone remodeling at any point on a bone surface, have been reported to be ~6 days for rats and 6–9 months for humans (Taguchi & Lopez, 2021). These differences have an important role in the study design and interpretation of the study data. Previous research in humans has investigated the effects of SARMs on lean body mass and bone mineral density (BMD) via dual energy x‐ray absorptiometry (DEXA) with intervention periods ranging from 21 days to 6 months (Basaria et al., 2013; Dalton et al., 2011; Papanicolaou et al., 2013). Increases in lean body mass were reported (Basaria et al., 2013; Dalton et al., 2011; Papanicolaou et al., 2013), but no difference was reported for BMD for up to 3 mg of enobosarm for 86 days (Dalton et al., 2011). Taking into account the differences in bone remodeling period between rats and humans, the age of the rats used (in growing phase), the sensitivity of the microCT, and the limitations to the safe duration of SARM treatment, it was determined that 2 weeks would be a relevant treatment duration. The effects of an FO model on bone remodeling are not well understood. Resistance training and FO models lead to increased circulating levels of various factors, some of which are likely released from the skeletal muscle and can be collectively referred to as a secretome (Leuchtmann et al., 2021). Some secretome factors, such as IGF‐I, are known to have a positive impact on bone mineralization and growth (Banu et al., 2003; Breen et al., 2011). Importantly, the full spectrum of the skeletal muscle resistance training‐induced secretome and their potential impact on bone remodeling, either alone or in combination, are not well understood (Leuchtmann et al., 2021). Our model provided ample opportunity to collect this measure while investigating our central hypothesis. After thoroughly analyzing the tibia microCT data, no statistically significant results were found between the four treatment groups for any of the measures.

Previous studies investigating the role of SARMs on skeletal muscle hypertrophy in rodents have investigated their ability to offset the loss of muscle mass in orchiectomized (ORX) rats (Gao, Reiser, et al., 2005). In this application, the SARMs are able to prevent the loss of soleus muscle mass; however, body mass is still reduced in the animals receiving SARM treatment. Therefore, there is a disconnect between the effects observed in certain skeletal muscles and the overall effect on body mass. Similarly, previous research has demonstrated that SARMs can increase lean body mass and bone mineral density while decreasing fat mass in orchiectomized rats (Jones et al., 2009). These effects can offset one another with respect to changes in body mass. Another aspect to consider is the potential for specific skeletal muscles to respond to SARMs in a manner that reflects AR content. The response observed in the hindlimb muscles and the response to RAD140 may not be the same for all muscles of the body as it is known that AR expression can vary greatly between different skeletal muscles (Antonio et al., 1999; Johansen et al., 2007; Kadi et al., 2000; Monks et al., 2006). Studies investigating SARMs in intact, non‐orchiectomized rats are rare. However, Donner et al. (Donner et al., 2015) investigated the impact of trenbolone on body composition in normogonadic rats. Trenbolone is a testosterone analog with SARM‐like actions due to its similar evasion of 5α‐reduction (Yarrow et al., 2011). A significant increase of ~8% in body mass was observed in control rats, while a nonsignificant decrease of ~5% in body mass was reported in the SARM‐like group. Despite the nonsignificant decrease in body mass in the SARM‐like group, there was a significant increase in lean body mass of ~50 g with a corresponding decrease in fat mass of ~81 g (Donner et al., 2015). Therefore, we did not expect an increase in body mass in the RAD140‐treated animals compared to the Vehicle. In the present study, RAD140 did cause an increase in soleus mass and plantaris muscle fiber CSA and mass; however, there were no differences in body weight between groups.

Although certain SARMs have undergone and are currently involved in clinical trials, the evaluation of SARMs has not yet reached the stage where the data have shown that potential therapeutic value outweighs the risks associated with their use. In a recent systematic review involving clinical trials evaluating the safety of SARMs, 1447 participants receiving SARM treatment were evaluated. While there was a calculated 7.1% mean across all studies of reported elevated alanine aminotransferase (ALT), the duration of SARM ranged from 21 days to 6 months (Vignali et al., 2023). In a phase 1 safety trial, Basaria et al. (2013) reported that none of the participants (76 healthy men) experienced elevated circulating liver enzymes following 21 days of treatment (Basaria et al., 2013). While the 21 days of the SARM LGD‐4033 did decrease total testosterone, sex hormone‐binding protein, and high‐density lipoprotein cholesterol, these were observed to return to baseline at 35 days after cessation of treatment (Basaria et al., 2013). Other clinical trials have reported that very few of the participants experienced elevated circulating liver enzymes following treatment durations ranging from 12 weeks to 6 months in frail elderly women (Papanicolaou et al., 2013), healthy elderly men, and postmenopausal women (Dalton et al., 2011), and male prostate cancer survivors (Pencina et al., 2021). Moreover, some of these studies reported that the elevated circulating liver enzymes were reversible and returned to baseline after discontinuation of the SARM use (Papanicolaou et al., 2013; Pencina et al., 2021). In a Phase 2 clinical trial investigating the activity and safety of the SARM enobosarm in the treatment of cancer, it was reported that there are benefits for use in the treatment of advanced breast cancer. However, several drug‐related adverse events were reported. Following 24 weeks of treatment, 8% and 16% of participants had grade 3 or grade 4 drug‐related adverse events for the low and high dose treatment groups, respectively. These drug‐related adverse events included increased hepatic transaminases (4% and 3% for low and high dose, respectively), hypercalcemia (3% and 3% for low and high dose, respectively), and fatigue (1% and 1% for low and high dose, respectively) (Palmieri et al., 2024). Based on the literature, it was not hypothesized that 14 days of RAD140 treatment in young healthy rats would have resulted in liver or cardiac injury; therefore, these samples were not collected and could not be evaluated.

Unfortunately, SARMs are currently being marketed and sold as dietary supplements or “sold for research use only”; however, they are unapproved drugs. The U.S. Food and Drug Administration has issued warnings to consumers of the potential dangers of using these drugs in an unregulated or approved manner (U.S. Food & Drug Administration, 2023). Case reports have also been published of self‐medicating users of RAD140 presenting with acute myocarditis (Padappayil et al., 2022) and liver injury (Demangone et al., 2024; Leung et al., 2022; Perananthan & George, 2024). Unregulated or recreational use that has been reported to cause negative impacts to health are difficult to track as the dosage and duration are not controlled. In addition, the source of the SARM could contain any number of other ingredients, and the reporting of side‐effects is not reliable. Any future studies that investigate the use of SARMS must properly track dosage, duration and the assessment of side effects. In addition, robust pre‐clinical safety studies should be conducted to evaluate the potential of SARMs to cause liver and cardiac damage. These studies must include the determination of potential safety limitation to both dosage and duration of treatment. There must be a clear risk assessment that balances the cost–benefit ratio for their intended use.

## 5 CONCLUSION

There are several populations that would benefit from therapeutic options to reduce muscle wasting, improve muscle mass and strength, while at the same time having a positive effect on bone mineralization. For example, sarcopenia is an age‐related loss of skeletal muscle mass and strength, with an estimated to affect ~10% of people >60 years of age. As populations age, so does the prevalence of sarcopenia (Beaudart et al., 2025; Shafiee et al., 2017). Similarly, the deterioration and loss of bone mass, osteoporosis, has been estimated to affect ~18% of the World's population (Salari et al., 2021) and has a negative impact on total joint arthroplasty (TJA) outcomes (Daher et al., 2024; Salari et al., 2021; So et al., 2021). While SARMs may pose a potential for therapeutic use, current information would indicate that safe treatment duration is limited. Therefore, future clinical use might be best suited to those who may benefit from a shorter treatment duration. For example, patients preparing for, or recovering from, TJA may benefit from limited duration SARM therapy. In the present study, it was determined that the administration of RAD140 resulted in a greater plantaris muscle fiber CSA compared to the Vehicle‐Control group, and plantaris muscle mass was higher in the RAD140‐FO compared to the RAD140 alone. In addition, the soleus muscle mass was greater in response to RAD140 treatment compared to the Vehicle‐Control. There were no significant findings in the tibia bone data; however, the rodent model used in the present study was young, healthy rats. Based on the potential therapeutic benefit of SARMs, future research should include investigation of their effects in combination with hypertrophy‐inducing exercise models in aging. Since RAD140 alone was comparable to the effect of either RAD140‐FO or FO alone on plantaris muscle mass and CSA, future research should also consider the ability of SARMs to offset loss of muscle mass and bone relevant to long‐term bed rest or space flight (e.g., hindlimb suspension model).

## AUTHOR CONTRIBUTIONS

Jake Puskas was involved in formal analysis, investigation, methodology, writing—original draft, and writing—review and editing. Teja Guda was involved in formal analysis and writing—review and editing. Sarah Niccoli was involved in investigation, methodology, and writing—review and editing. Christopher Rathbone was involved in conceptualization, methodology, and writing—review and editing. Benjamin Tan‐Johnson was involved in, formal analysis, investigation, and writing—review and editing. David Puskas was involved in conceptualization, funding acquisition, project administration, and writing—review and editing. Ryan Middleton was involved in formal analysis, investigation, writing—review and editing. Jeffrey S. Otis was involved in conceptualization, formal analysis, investigation, methodology, project administration, resources, supervision, and writing—review and editing. Simon J. Lees was involved in conceptualization, formal analysis, funding acquisition, investigation, methodology, project administration, resources, supervision, writing—original draft, writing—review and editing.

## FUNDING INFORMATION

This research was supported by a Northern Ontario Academic Medicine Association AFP Innovation Funds grant (PI: Dr. David Puskas), Northern Ontario School of Medicine University Research and Development Grant (PI: Dr. Simon J. Lees).

## CONFLICT OF INTEREST STATEMENT

The authors declare that the research was conducted in the absence of any commercial or financial relationships that could be construed as a potential conflict of interest.

## ETHICS STATEMENT

All procedures were approved by the Lakehead University Animal Care Committee and the Georgia State University Institutional Animal Care and Use Committee.

## Data Availability

The data that support the findings of this study are available from the corresponding author (SJL), upon reasonable request.
